# The Role of Metabolomics in Understanding Hypertension: Protocol for a Systematic Review and Meta-Analysis

**DOI:** 10.2196/77536

**Published:** 2026-03-17

**Authors:** Mohd Naeem Mohd Nawi, Puteri Sofia Nadira Megat Kamaruddin

**Affiliations:** 1 Nutrition, Metabolism and Cardiovascular Research Centre, Institute for Medical Research, National Institutes of Health, Ministry of Health Malaysia, No.1, Jalan Setia Murni U13/52, Seksyen U13, Setia Alam, Shah Alam, 40170, Malaysia, 60 33627840

**Keywords:** hypertension, metabolomics, biomarkers, profiling, diagnosis

## Abstract

**Background:**

Hypertension is a chronic condition and a leading risk factor for cardiovascular disease, stroke, and premature mortality worldwide. While blood pressure (BP) monitoring—via clinical, home, or ambulatory measurements—remains the primary diagnostic tool, each method is limited by variability, device inaccuracy, and difficulties in detecting atypical BP patterns such as masked or white-coat hypertension. These challenges underscore the need for innovative, complementary diagnostic approaches.

**Objective:**

This systematic review and meta-analysis aims to synthesize current evidence on the role of metabolomic profiling in the detection and understanding of hypertension. Specifically, it seeks to evaluate whether metabolomics can identify preclinical metabolic signatures, improve diagnostic accuracy, and elucidate pathophysiological mechanisms underlying hypertension, thereby complementing traditional BP monitoring.

**Methods:**

A systematic search will be conducted in PubMed, Embase, Web of Science, Scopus, and CENTRAL from inception to the present supplemented with manual screening of metabolomics repositories (MetaboLights and Metabolomics Workbench) to ensure comprehensive coverage. This systematic review will include studies involving adults (aged ≥18 years) that investigate metabolomic biomarkers in hypertension using validated analytical platforms such as nuclear magnetic resonance spectroscopy, gas chromatography–mass spectrometry, or liquid chromatography–mass spectrometry. Eligible studies must stratify participants into hypertensive, prehypertensive, or normotensive groups and report associations between metabolites and BP measurements. Two independent reviewers will conduct study selection, data extraction, and risk-of-bias assessment using version 2 of the Cochrane risk-of-bias tool for randomized trials and the Risk of Bias in Nonrandomized Studies of Interventions and Risk of Bias in Nonrandomized Studies of Exposures tools, with evidence certainty evaluated via the Grading of Recommendations Assessment, Development, and Evaluation framework. A meta-analysis will be performed where possible using random-effects models and subgroup analyses to address heterogeneity.

**Results:**

Database search, screening, and data extraction are ongoing as of January 2026. The results will summarize metabolomic profiles (such as stearidonate and hexadecadienoate), diagnostic accuracy metrics (eg, area under the curve and sensitivity), and metabolic pathways associated with hypertension once data synthesis is completed. The systematic review and meta-analysis is expected to be published in August 2026.

**Conclusions:**

This review will identify metabolites showing consistent hypertension associations and document methodological requirements for clinical translation, including standardization of sample collection, analytical platforms, and data processing. The findings will inform longitudinal validation studies and establish evidence-based pathways for potential applications in hypertension risk assessment and clinical trial design. Validation of consistently identified metabolite-hypertension associations in independent, prospective cohorts will be critical for establishing causality and assessing the temporal stability of metabolomic signatures. The review will also identify methodological gaps requiring standardization, such as sample collection protocols, analytical platform harmonization, and data processing procedures, which must be addressed before metabolomic findings can be translated into clinical applications.

## Introduction

High blood pressure (BP), or hypertension, is defined by sustained elevations in arterial pressure and is recognized as a leading risk factor for cardiovascular disease, stroke, and premature mortality worldwide [1]. BP monitoring remains the primary method for detecting hypertension using office-based (measurements taken in a clinical setting during a scheduled visit), home, and ambulatory measurements [1]. However, each approach presents notable challenges. Office BP measurement, while widely used, is susceptible to variability due to factors such as improper technique, patient anxiety, and time constraints, which can lead to inaccurate readings and misclassification of hypertension status [2,3]. Additionally, office measurements often fail to detect masked hypertension, in which BP is normal in the office but increased elsewhere, and white-coat hypertension, in which BP is elevated in the clinical setting but normal elsewhere, both of which are prevalent and clinically significant [2,4,5]. Home BP monitoring can help identify these phenomena but depends heavily on patient adherence to proper measurement protocols and the accuracy of devices, many of which are not validated [6,7]. Ambulatory BP monitoring is widely regarded as the most reliable method for diagnosing hypertension, especially for identifying cases of white-coat and masked hypertension, as well as for assessing nocturnal BP patterns and BP variability, which are strong predictors of cardiovascular risk [5,8]. Nevertheless, ambulatory BP monitoring is less accessible, can be poorly tolerated, and is subjected to measurement artifacts that affect reproducibility [3,5]. In summary, while BP monitoring is essential for hypertension detection, its inherent limitations, including measurement variability, device inaccuracy, and challenges in identifying atypical BP patterns, highlight the need for continued innovation and complementary diagnostic strategies to improve detection and management.

Metabolomics, the study of small-molecule metabolites (<1500 Da) in biological systems, provides dynamic insights into physiological states by analyzing pathways such as lipid metabolism and amino acid turnover [9]. Unlike genomic or proteomic approaches, it captures real-time metabolic changes influenced by genetics, environment, and lifestyle, making it pivotal for understanding noncommunicable diseases (NCDs) [10,11]. NCDs, responsible for approximately 74% of global mortality, are linked by shared metabolic disruptions identified through metabolomics, such as altered glycerophospholipid metabolism and mitochondrial dysfunction markers, which underlie conditions such as diabetes and obesity [11,12]. For instance, branched-chain amino acids and ceramides are elevated across metabolic and cardiovascular disorders, serving as cross-disease biomarkers linked to insulin resistance and inflammation [11,13]. Metabolomics additionally uncovers metabolites produced from the gut microbiota, including trimethylamine N-oxide, that contribute to systemic inflammation and renal or hepatic complications in NCDs [14]. Clinically, metabolomic profiling enables early risk prediction—detecting metabolic signatures years before diagnosis—and guides personalized interventions such as micronutrient supplementation or mitochondrial-targeted therapies, improving outcomes such as quality-adjusted life years [10,13]. By mapping these pathways, metabolomics complements traditional diagnostics, offering a proactive approach to managing NCD multimorbidity.

While metabolomics holds considerable promise for advancing hypertension research, existing literature reveals critical gaps that underscore the need for rigorous synthesis of available evidence. The most recent comprehensive narrative review examined studies published between 2015 and 2025, identifying key disrupted metabolic pathways, including amino acid metabolism (particularly branched-chain and aromatic amino acids), fatty acid metabolism (ceramides, oleic acid, and short-chain fatty acids), and pathways related to inflammation and oxidative stress [15,16]. However, this review did not use systematic methodology or conduct meta-analyses to quantify the magnitude of metabolite alterations or evaluate their diagnostic performance characteristics such as sensitivity, specificity, or area under the curve (AUC).

This systematic review and meta-analysis aims to synthesize evidence on the utility of metabolomic profiling in detecting hypertension and complementing BP monitoring by identifying preclinical metabolic signatures, improving diagnostic accuracy, and elucidating pathophysiological mechanisms. This systematic review will evaluate metabolomics’ capacity to address limitations of traditional BP measurement, such as episodic variability, white-coat hypertension, and delayed diagnosis, through objective biomarker discovery.

## Methods

### Eligibility Criteria

#### Overview

This review focuses on studies that investigate metabolomic biomarkers specifically in relation to hypertension detection and metabolic mechanisms underlying BP regulation, including any linkage such as correlations or diagnostic accuracy metrics (AUC and sensitivity) and excluding broad exploratory or non–hypertension-specific metabolomic analyses. This systematic review will include studies that investigate human participants aged 18 years or older regardless of sex or ethnicity who have been diagnosed with hypertension (systolic BP [SBP]≥130 mm Hg or diastolic BP≥80 mm Hg), prehypertension (SBP of 120‐129 mm Hg), or normal BP levels. Eligible studies must use metabolomic profiling techniques such as nuclear magnetic resonance (NMR) spectroscopy, gas chromatography–mass spectrometry (GC-MS), or liquid chromatography–mass spectrometry (LC-MS) to identify metabolites or metabolic pathways associated with hypertension. Only original research articles with clearly defined study designs (eg, case-control, cohort, or cross-sectional) will be considered. Exclusion criteria include animal studies, nonoriginal research (reviews and editorials), studies lacking primary metabolomic or BP data, populations with comorbidities (eg, diabetes or chronic kidney disease), pregnancy, or use of confounding medications.

Studies will be selected based on predefined inclusion and exclusion criteria to ensure methodological consistency and relevance to the research objectives. The criteria were developed using the population, intervention or exposure, comparator, and outcomes framework and aligned with the overall aim of synthesizing metabolomic evidence in hypertension. To enhance readability, the inclusion and exclusion criteria are summarized in Textbox 1.

Textbox 1. **Inclusion and exclusion criteria.**

**Inclusion criteria**

- Human studies involving adults aged ≥18 years
- Studies investigating metabolomic biomarkers in hypertension
- Use of validated analytical platforms (eg, nuclear magnetic resonance spectroscopy, gas chromatography–mass spectrometry, or liquid chromatography–mass spectrometry)
- Stratification of participants into hypertensive, prehypertensive, or normotensive groups
- Reporting of associations between metabolites and blood pressure or diagnostic metrics (eg, area under the curve, sensitivity, and specificity)

**Exclusion criteria**

- Animal or in vitro studies
- Reviews, editorials, conference abstracts without full data, or nonoriginal research
- Studies lacking primary metabolomic or blood pressure data
- Populations with major comorbidities (eg, diabetes or chronic kidney disease), pregnancy, or use of confounding medications
- Studies not meeting the aforementioned inclusion criteria

#### Population

The target population includes adults aged 18 years and older stratified into hypertensive, prehypertensive, and normotensive groups. Studies involving secondary hypertension, acute illness, or metabolic disorders will be excluded to minimize confounding effects on metabolomic profiles that may arise from conditions such as diabetes or chronic kidney disease. Where eligible studies include mixed populations, data pertaining to primary hypertensive subgroups or sensitivity analyses will be extracted and analyzed separately where possible to maintain relevance and improve generalizability.

#### Intervention

The intervention or exposure involves conducting metabolomic profiling through techniques such as NMR spectroscopy, GC-MS, or LC-MS, with the aim of identifying biomarkers that are associated with hypertension.

#### Comparison

Comparisons will be made between individuals with hypertension and normal BP, prehypertensive and normotensive groups, and individuals before or after antihypertensive treatment.

#### Outcome Measures

Primary outcomes include diagnostic accuracy of metabolomic biomarkers (eg, AUC and sensitivity) and identification of key metabolites linked to hypertension (eg, stearidonate and hexadecadienoate). Secondary outcomes include pathway dysregulation (eg, lipid peroxidation and mitochondrial dysfunction) and correlations between metabolite levels and BP changes.

### Data Sources, Search Terms, and Search Strategy

This systematic review will use 5 electronic databases—PubMed, Embase, Web of Science, Scopus, and CENTRAL—from inception to the present as primary data sources. These databases were selected to ensure broad coverage of biomedical and omics-related literature. In addition, specialized metabolomics repositories such as MetaboLights and Metabolomics Workbench will be manually searched to identify relevant studies and supplementary datasets not indexed in the aforementioned databases. Both authors collaborated to determine relevant search terms, including keywords and MeSH (Medical Subject Headings) terms, and search queries were constructed using Boolean operators (“AND” and “OR”). Searches will be conducted from the inception of each database to the most recent available studies without applying search filters. No language restrictions will be applied during the search. In cases in which potentially relevant articles are published in languages other than English, professional and credible translation services will be used to translate the texts into English for inclusion and assessment. Unpublished studies identified at the time of the final search will be excluded. The detailed search strategy is shown in Multimedia Appendix 1. All retrieved references will be downloaded in RIS format and managed using EndNote (version 19; Clarivate Analytics), which will be used to combine records from all databases and remove duplicates.

### Study Screening and Selection

The screening of eligible studies will be undertaken after the database searches are complete. All identified references will be managed using Microsoft Excel, where duplicates will be identified and removed. To ensure consistency and familiarity with the screening process, at least 5 randomly selected studies will be pilot-tested by the reviewers. The evaluation will occur in 2 stages. Initially, the 2 reviewers (MNMN and PSNMK) will independently evaluate the titles and abstracts of all references based on the established eligibility criteria. During the second stage, the same 2 reviewers will evaluate the complete texts of possibly suitable articles for inclusion. Disagreements or differences between the reviewers will be resolved through discussion to achieve consensus. This methodology aims to reduce bias and guarantee a transparent and methodical selection of studies. We will document the selection process in adequate detail to finalize a PRISMA (Preferred Reporting Items for Systematic Reviews and Meta-Analyses) flow diagram [16].

### Data Extraction

Data extraction will use a standardized Microsoft Excel template to record analytical platforms (NMR spectroscopy, GC-MS, and LC-MS); quantified metabolites (such as amino acids and lipids); associated metabolic pathways; and statistical associations with BP parameters, including SBP or diastolic BP values and diagnostic accuracy metrics. The template will systematically capture study characteristics, including design, population demographics, interventions, comparators, and outcomes related to metabolite-BP linkages. The 2 reviewers (MNMN and PSNMK) will independently extract data from the selected papers, and any disagreements will be resolved through consensus. If further clarification or information is required, the corresponding authors of the original studies will be contacted.

### Risk-of-Bias and Quality Assessment

For randomized controlled trials, the risk-of-bias assessment will use version 2 of the Cochrane risk-of-bias tool for randomized trials; for nonrandomized studies, the Risk of Bias in Nonrandomized Studies of Interventions tool will be applied. For observational studies examining metabolomic exposures and hypertension outcomes, which represent most metabolomics research designs, we will apply the Risk of Bias in Nonrandomized Studies of Exposures (ROBINS-E) tool [17]. The ROBINS-E tool is specifically designed for cohort and case-control studies evaluating biological exposures such as metabolite concentrations, assessing 7 bias domains: confounding, participant selection, exposure classification, deviations from intended exposures, missing data, outcome measurement, and selective reporting. To address metabolomics-specific methodological challenges within the ROBINS-E framework, we will systematically evaluate (1) preanalytical factors, including sample collection protocols, storage conditions, freeze-thaw cycles, and metabolite stability considerations; (2) analytical quality control procedures, including use of pooled quality control samples, internal standards, coefficient of variation reporting, and batch effect correction methods; (3) metabolite identification confidence according to Metabolomics Standards Initiative criteria (levels 1-4); and (4) data processing rigor, including normalization, missing value handling, and multiple testing correction. The 2 reviewers (MNMN and PSNMK) will independently assess each study, and any differences in their assessments will be resolved through discussion. If essential information is unclear or unavailable in the published reports, additional details will be sought by contacting the study investigators. This rigorous approach ensures a transparent and systematic evaluation of study quality and potential sources of bias. The certainty of evidence for each outcome will be assessed using the Grading of Recommendations Assessment, Development, and Evaluation approach, and a summary table will be presented, including justifications for each assessment.

### Data Synthesis

A meta-analysis will be conducted if 2 or more studies provide identical outcomes; otherwise, the results will be described narratively. For metabolomics studies, “identical outcomes” will be defined as the same metabolite confirmed through International Chemical Identifier Key [18] matching, ensuring consistent identification across studies regardless of naming conventions, with cross-referencing through PubChem [19], the Human Metabolome Database [20], the Kyoto Encyclopedia of Genes and Genomes [21], and the Reference Set of Metabolite Names [22] to resolve nomenclature discrepancies. Data analysis will use The Cochrane Collaboration’s Review Manager software (version 5.4) for aggregating results. Continuous outcomes will be computed as mean differences or standardized mean differences with 95% CIs, depending on the consistency of the measuring instruments used across studies. Dichotomous outcomes will be represented as odds ratios accompanied by 95% CIs. The *I*^2^ statistic will assess heterogeneity, with values of 0% to 40% indicating low heterogeneity, 30% to 60% indicating moderate heterogeneity, 50% to 90% indicating substantial heterogeneity, and 75% to 100% indicating considerable heterogeneity. Analyses will use random-effects models to account for heterogeneity, particularly when pooling across different metabolomics methodologies.

Given the inherent variability in metabolomics platforms (NMR spectroscopy vs LC-MS vs GC-MS) and metabolite panels, meta-analyses will be stratified by analytical platform type, with platform-specific pooled estimates calculated separately before assessing cross-platform comparability using subgroup heterogeneity tests (Cochran *Q* statistic; interaction *P*<.10). When substantial between-platform heterogeneity exists (*I*^2^>75% or significant platform interaction), results will be presented separately by platform rather than pooled across methodologies. Studies will be documented for their analytical platform specifications (instrument type, column chemistry for LC-MS, and field strength for NMR spectroscopy), and these characteristics will inform quality weighting in meta-analyses. Sensitivity analyses will be performed by (1) excluding each analytical platform sequentially to assess platform-specific influence on pooled estimates; (2) comparing targeted vs untargeted metabolomics approaches; (3) restricting analyses to studies using validated, standardized protocols; (4) excluding studies with high risk of bias; and (5) restricting analyses to studies with Metabolomics Standards Initiative level 1 and 2 metabolite identifications. Meta-regression will be performed when at least 10 studies are available, with analytical platform type, sample matrix (serum vs plasma vs urine), and study quality as moderator variables to quantify their contribution to heterogeneity. Predefined subgroups will include age, sex, geographic region, analytical platform (NMR spectroscopy vs LC-MS vs GC-MS), sample type (serum vs plasma vs urine), metabolite identification confidence level (levels 1-2 vs 3-4), study design (cross-sectional vs prospective cohort), and risk-of-bias categories. Assessment of publication bias will be conducted using funnel plot symmetry and the Egger regression approach when a significant number of papers (generally ≥10) are present. For outcomes characterized by inadequate data, an *I*^2^ value of more than 75%, significant platform-related heterogeneity, or fewer than 2 studies reporting identical metabolites, or when cross-platform differences preclude reliable pooling, findings will be synthesized narratively with results stratified by analytical platform and metabolite class. This comprehensive approach ensures that metabolomics-specific methodological variability is systematically addressed through predefined strategies, enhancing the reliability and interpretability of pooled estimates.

### Registration and Reporting

This protocol has been registered with PROSPERO (CRD420251016289). Any protocol amendments will be documented on the PROSPERO record, including the date, description, and rationale for each change. The review will adhere to the PRISMA 2020 statement [23] and PRISMA-P (Preferred Reporting Items for Systematic Reviews and Meta-Analyses Protocols) [24] guidelines and will be reported according to the *Cochrane Handbook for Systematic Reviews of Interventions* [25]. The PRISMA 2020 checklist has been provided (Checklist 1).

## Results

**Figure 1.**
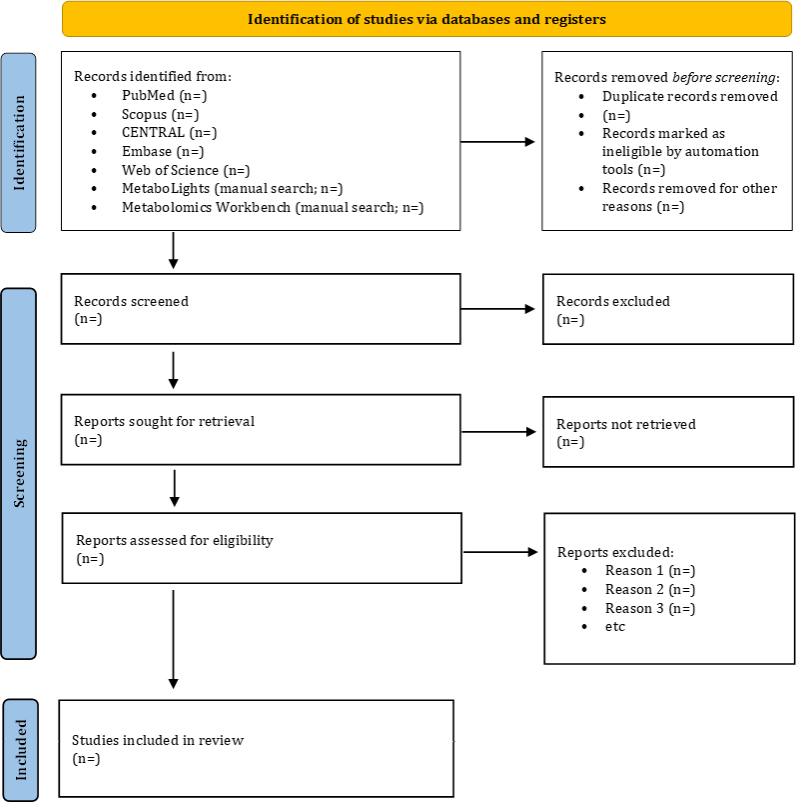
PRISMA (Preferred Reporting Items for Systematic Reviews and Meta-Analyses) flow diagram.

The database search, screening, and data extraction and synthesis are ongoing as of January 2026. The results of this systematic review and meta-analysis will summarize diagnostic accuracy metrics, key metabolites, and metabolic pathways associated with hypertension once the analysis is completed. The systematic review and meta-analysis is expected to be published in August 2026. Figure 1 shows the PRISMA flow diagram.

## Discussion

### Overview

There is increasing recognition of the important role that metabolomics can play in advancing the understanding, detection, and management of hypertension, yet large-scale, systematic data on its application in hypertensive populations remain limited. Comprehensive studies investigating metabolomic signatures and their association with BP are critical for elucidating the underlying mechanisms of hypertension and informing the development of more precise diagnostic and therapeutic strategies.

A central hypothesis in this field is that a combination of genetic, environmental, and lifestyle factors, particularly in vulnerable populations such as those with lower socioeconomic status or coexisting metabolic disorders, may contribute to distinct metabolomic profiles associated with increased hypertension risk [26]. Additionally, variability in the accuracy and validity of different metabolomic techniques may result in inconsistent detection of relevant biomarkers, potentially leading to underestimation or overestimation of hypertension risk. The interplay of multiple contextual risk factors and methodological differences could jointly influence the observed associations between metabolomic markers and hypertension [27].

This systematic review and meta-analysis will synthesize the available literature to clarify the relationship between metabolomic profiles and hypertension, including the prevalence and distribution of key biomarkers; the influence of risk factors such as age, sex, ethnicity, and comorbidities; and the impact of different detection methods on study outcomes. By integrating data from diverse populations and analytical platforms, this review aims to provide a more nuanced and comprehensive understanding of the role of metabolomics in hypertension. The findings are expected to highlight that factors such as socioeconomic status, lifestyle, and methodological limitations in metabolite detection are key barriers to the effective clinical translation of metabolomics in hypertension. Furthermore, the review will provide a transferable framework for the application of metabolomics in hypertension research and practice.

### Limitations

A limitation of this review is that data on metabolomic profiles in hypertension are often derived from secondary analyses of clinical or epidemiological datasets, which may be subjected to incomplete reporting or variability in sample handling and analytical protocols. While such data reflect real-world clinical practice, they may also introduce bias or limit the generalizability of the findings. Implementing a model that requires prospective, standardized metabolomic assessments may not be feasible in all health care settings, particularly where resources or expertise are limited. It is important to recognize that the proposed framework is preliminary and should not be directly applied in clinical practice without further validation. Another limitation is the potential for selection bias, as studies may vary in the timing of sample collection relative to disease onset or treatment and may exclude individuals with acute or secondary forms of hypertension, thereby restricting the applicability of the findings to the broader hypertensive population. This review is also limited by the exclusion of participants with major comorbidities or secondary hypertension, potentially reducing generalizability to real-world hypertensive populations. This criterion was applied to isolate metabolomic signatures specific to primary hypertension, and future research including comorbid groups will be needed to assess external validity.

### Conclusions

The findings from this systematic review will inform future research directions by identifying priority areas for investigation, including the need for longitudinal studies that incorporate serial metabolomic assessments to capture temporal changes across different stages of hypertension development and progression. Validation of consistently identified metabolite-hypertension associations in independent, prospective cohorts will be critical for establishing causality and assessing the temporal stability of metabolomic signatures. The review will also identify methodological gaps requiring standardization, such as sample collection protocols, analytical platform harmonization, and data processing procedures, which must be addressed before metabolomic findings can be translated into clinical applications.

The clinical integration of metabolomics into hypertension management will require a staged approach informed by the evidence synthesized in this review. On the basis of the strength and consistency of the findings, several translational pathways can be envisioned. If the review identifies metabolites with robust, replicated associations with hypertension across multiple studies and platforms, these could be incorporated into research-focused risk assessment tools or used to stratify participants in clinical trials of antihypertensive interventions. This would not require changes to clinical guidelines but would enhance research efficiency and mechanistic understanding. Metabolite panels showing consistent associations with hypertension could be evaluated in targeted validation studies comparing their additive predictive value beyond conventional risk factors. If validated metabolite panels demonstrate clinically meaningful improvements in risk prediction (eg, reclassification of 10%-15% of individuals from intermediate to high- or low-risk categories), they could be considered for incorporation into hypertension screening algorithms for high-risk populations, similar to how lipid panels are used in cardiovascular risk assessment.

## Supplementary material

10.2196/77536 Multimedia Appendix 1 Search strategy.

10.2196/77536 Checklist 1 PRISMA 2020 checklist.

## Abbreviations

AUC: area under the curve
BP: blood pressure
GC-MS: gas chromatography–mass spectrometry
LC-MS: liquid chromatography–mass spectrometry
MeSH: Medical Subject Headings
NCD: noncommunicable disease
NMR: nuclear magnetic resonance
PRISMA: Preferred Reporting Items for Systematic Reviews and Meta-Analyses
PRISMA-P: Preferred Reporting Items for Systematic Reviews and Meta-Analyses Protocols
ROBINS-E: Risk of Bias in Nonrandomized Studies of Exposures
SBP: systolic blood pressure
