# Supplementary material for: The Role of Metabolomics in Understanding Hypertension: Protocol for a Systematic Review and Meta-Analysis
Source: JMIR Res Protoc. 2026 Mar 17;15:e77536. doi: 10.2196/77536 (PMC12994758; doi:10.2196/77536)
Supplement: Multimedia Appendix 1 [file resprot-v15-e77536-s001.docx]

| **DATABASE** | **SEARCH STRATEGY** |
| --- | --- |
| Cochrane Central Register of Controlled Trials (CENTRAL) | "Adult" OR "Young Adult" OR "Middle Aged" OR "Man" OR "Woman" OR "Men" OR "Women" OR "Patient" OR "Person" OR “Human” OR "Individual" in Title Abstract Keyword AND "metabolomics" in Title Abstract Keyword AND "liquid chromatography-mass spectrometry" OR "LC-MS" OR "LCMS" OR "LC/MS" OR "gas chromatography-mass spectrometry" OR "GC-MS" OR "GCMS" OR "GC/MS" OR "nuclear magnetic resonance" OR "NMR" OR "proton NMR" OR "1H NMR" OR "chromatography" OR "mass spectrometry" in Title Abstract Keyword AND "hypertension" OR "high blood pressure" OR "elevated blood pressure" in Title Abstract Keyword |
| Embase | 1. (adult/ OR young adult/ OR middle aged/ OR man/ OR woman/ OR patient/ OR human/ OR exp adult/)  2. (adult OR "young adult" OR "middle aged" OR man OR woman OR men OR women OR patient OR person OR human OR individual).ti,ab,kw.  3. 1 OR 2  4. metabolomics/ OR metabolome/  5. (metabolomic* OR metabolome*).ti,ab,kw.  6. 4 OR 5  7. exp liquid chromatography mass spectrometry/ OR exp gas chromatography mass spectrometry/ OR exp nuclear magnetic resonance spectroscopy/ OR exp chromatography/ OR exp mass spectrometry/  8. ("liquid chromatography-mass spectrometry" OR LC-MS OR LCMS OR "LC/MS" OR "LC MS" OR "gas chromatography-mass spectrometry" OR GC-MS OR GCMS OR "GC/MS" OR "GC MS" OR "nuclear magnetic resonance" OR NMR OR "proton NMR" OR "1H NMR" OR "1H-NMR" OR chromatography OR "mass spectrometry").ti,ab,kw.  9. 7 OR 8  10. exp hypertension/  11. (hypertension OR "high blood pressure" OR "elevated blood pressure" OR "raised blood pressure" OR "increased blood pressure" OR hypertensive).ti,ab,kw.  12. 10 OR 11  13. 3 AND 6 AND 9 AND 12  14. limit 13 to (human and english language) |
| Scopus | ("Adult" OR "Young Adult" OR "Middle Aged" OR "Man" OR "Woman" OR "Men" OR "Women" OR "Patient" OR "Person" OR "Human" OR "Individual") AND ("metabolomics" ) AND ("liquid chromatography-mass spectrometry" OR "LC-MS" OR "LCMS" OR "LC/MS" OR "gas chromatography-mass spectrometry" OR "GC-MS" OR "GCMS" OR "GC/MS" OR "nuclear magnetic resonance" OR "NMR" OR "proton NMR" OR "1H NMR" OR "chromatography" OR "mass spectrometry") AND ("hypertension" OR "high blood pressure" OR "elevated blood pressure") |
| PubMed | ((("Adult"[Title/Abstract] OR "Young Adult"[Title/Abstract] OR "Middle Aged"[Title/Abstract] OR "Man"[Title/Abstract] OR "Woman"[Title/Abstract] OR "Men"[Title/Abstract] OR "Women"[Title/Abstract] OR "Patient"[Title/Abstract] OR "Person"[Title/Abstract] OR "Human"[Title/Abstract] OR "Individual"[Title/Abstract]) AND (metabolomics[Title/Abstract])) AND ("liquid chromatography-mass spectrometry"[Title/Abstract] OR "LC-MS"[Title/Abstract] OR "LCMS"[Title/Abstract] OR "LC/MS"[Title/Abstract] OR "gas chromatography-mass spectrometry"[Title/Abstract] OR "GC-MS"[Title/Abstract] OR "GCMS"[Title/Abstract] OR "GC/MS"[Title/Abstract] OR "nuclear magnetic resonance"[Title/Abstract] OR "NMR"[Title/Abstract] OR "proton NMR"[Title/Abstract] OR "1H NMR"[Title/Abstract] OR "chromatography"[Title/Abstract] OR "mass spectrometry"[Title/Abstract])) AND ("hypertension"[Title/Abstract] OR "high blood pressure"[Title/Abstract] OR "elevated blood pressure"[Title/Abstract]) |
| Web of Science | TS=((adult OR "young adult" OR "middle aged" OR man OR woman OR men OR women OR patient OR person OR human OR individual) AND (metabolomic* OR metabolome*) AND ("liquid chromatography-mass spectrometry" OR "LC-MS" OR LCMS OR "LC/MS" OR "LC MS" OR "gas chromatography-mass spectrometry" OR "GC-MS" OR GCMS OR "GC/MS" OR "GC MS" OR "nuclear magnetic resonance" OR NMR OR "proton NMR" OR "1H NMR" OR "1H-NMR" OR chromatography OR "mass spectrometry") AND (hypertension OR "high blood pressure" OR "elevated blood pressure" OR "raised blood pressure" OR "increased blood pressure" OR hypertensive)) |
